# A Global Analysis of Within-Country Health Inequalities

**DOI:** 10.1001/jamahealthforum.2025.3611

**Published:** 2025-10-17

**Authors:** Eran Bendavid, Allie K. S. Littleton, Iván Mejía-Guevara, Meredith Fenyo, Grant Miller

**Affiliations:** 1 Department of Medicine, Stanford University, Stanford, California; 2 Department of Health Policy, Stanford University, Stanford, California; 3 Biomedical Data Science, Stanford University, Stanford, California; 4 Consultant in Population Health Science, Palo Alto, California; 5 Care Yaya, Research Triangle Park, North Carolina; 6 Freeman Spogli Institute, Stanford University, Stanford, California; 7 Stanford Institute for Economic Policy Research, Stanford University, Stanford, California

## Abstract

**Importance:**

Health inequalities are a defining social and policy concern. However, understanding whether a specific country’s inequalities are large or small is limited without a comparative perspective with other countries.

**Objective:**

To systematically compare health inequalities in 181 countries and territories from 1960 to 2021 by developing a Health Inequality Normalized Index (HINI) that does not rely on secondary variables such as income, education, or race and ethnicity.

**Design, Setting, and Participants:**

This repeated cross-sectional study including demographic cross-national comparative analysis used age-at-death distributions from life tables for 181 countries and territories from 1960 to 2021. All county and territory health inequalities were ranked, and a random forest analysis was conducted of 191 factors to identify their relative role. Special attention was given to trends in health inequalities in the US. Data were analyzed between October 2023 and January 2025.

**Exposures:**

HINI was constructed by placing observed age-at-death distributions between perfect equality (conceptualized as everyone living exactly to the most common age at death) and the worst possible state of inequality (the largest variance in age-at-death distribution observed for each country).

**Main Outcomes and Measures:**

The primary outcome was the HINI, which measures within-country inequality in age-at-death distribution consistently for all countries and territories. Additional analyses explored the importance of potential factors (eg, infant mortality, wealth inequality, governance quality) associated with health inequalities.

**Results:**

Of 181 countries and territories between 1960 and 2021, in 2019 (pre–COVID-19), Turkmenistan had the highest HINI (most unequal), while Hong Kong had the lowest (most equal). Random forest analysis revealed that infant mortality and life expectancy were the primary factors associated with cross-country variation in HINI. Globally, health inequality decreased from 1960 to 2021, consistent with improvements in infant mortality and life expectancy. However, inequality trends diverged by country income group, improving more rapidly in high-income than in low-income countries. In the US, health inequality decreased but less than in other high-income nations: it ranked 19th in 1960 but 77th in 2021 among 181 countries and territories, and 55th among 59 high-income countries.

**Conclusions and Relevance:**

Results of this study suggest that infant mortality and life expectancy are critical factors in shaping countries’ health inequalities. Sustained improvements in countries with high infant mortality may have the potential to further reduce inequality at a global scale. In the US, comparatively slow progress on health inequality underscores its salience in national health policy discussions.

## Key Points

**Question:**

How do health inequalities in the US compare with health inequalities in other countries, and what changes health inequalities across countries or over time?

**Findings:**

In this repeated cross-sectional study involving an index of health inequality comparable across 181 countries and territories, on average, health inequalities were found to have decreased between 1960 and 2021. In the US, health inequality also decreased but less than in other high-income nations: it ranked 19th in 1960 but 77th in 2021 among 181 countries and territories, and 55th among 59 high-income countries.

**Meaning:**

In this study, infant mortality and life expectancy dominate factors associated with health inequality, and future improvements in countries with high infant mortality have potential to meaningfully improve health inequality.

## Teaser

This observational study including demographic cross-national comparative analysis constructs a unidimensional measure of health inequality grounded in a conceptual approach to health inequalities and characterizes the global distribution of country-level health inequalities from 1960 to 2021.

## Introduction

Inequalities and disparities in health and health care are a defining concern of public health and health policy, made more acute during the COVID-19 pandemic.^1,2,3,4^ A substantial amount of health policy and epidemiology research aims to identify health inequalities and the levers that can be used to reduce them.^5,6^ This is particularly true in the US, where the number of publications and National Institutes of Health research grants focused on health disparities have increased exponentially between 2004 and 2024.^7^

However, because no country has achieved perfect equality in health status, the import of any single country’s inequalities depends on comparisons with other countries or over time. Are health inequalities in the US better or worse than in other high-income countries? Are health inequalities getting better, staying the same, or getting worse over time? Where in the world are health disparities greatest, and where are they smallest? To the extent that health inequalities are undesirable (all else equal) and a possible target for interventions, a comparative view can guide prioritization and help with understanding the variables associated with health disparities on a global scale.

The importance of a comparative view may seem intuitive, and yet comparative analyses of within-country health inequality are rare for a few reasons. Health inequalities can be defined as differences between groups in a health measure of interest, such as differences in average life expectancy between different racial and ethnic groups. Commonly, and especially in the US, health inequalities are defined as differences in health measures along a second dimension, such as differences in life expectancy by income, race, or education.^8,9,10^

However, second dimensions such as income or race and ethnicity are not readily comparable across countries, posing a challenge for global perspectives on health inequalities.^11,12^ For instance, reporting differences in life expectancy among Hispanic, non-Hispanic Black, and non-Hispanic White populations may be valuable for the US but cannot be used where racial and ethnic group constructs are different. Similar limitations exist even for measures such as income or education, because social and economic contexts (or, for example, secondary school or top 10% of incomes) vary meaningfully across locations or are not well measured.^13,14^ The context-dependence of common second dimension socioeconomic stratifiers is a challenge for global comparative studies of health inequality.^15,16,17^

Assessing health inequalities along a single health dimension can relax these concerns and allow for cross-country comparisons. Several measures of univariate health inequality have been developed, but their use has been limited in the health inequalities literature. A Gini-like coefficient was developed and applied to assess inequality in age at death in England and Wales between 1931 and 1983.^18,19,20^ This Gini-like approach has been adopted for use with life table information.^21,22,23^ A similar index that compared inequalities in mortality with an ideal equality in mortality was developed by Jamison et al.^24^

The limited availability of univariate indices for comparing within-country health inequalities is a challenge for prioritizing action to address current levels of inequalities. Even in countries such as the US where health inequalities are researched, the extent to which inequalities are large or small is hard to assess without comparison benchmarks. This project aims to fill this knowledge gap by constructing a unidimensional measure of health inequality, the Health Inequality Normalized Index (HINI), that is grounded in a conceptual approach to health inequalities, and then characterizing the global distribution of country-level health inequalities, analyzing the variables associated with health inequalities. We also focus on US health inequalities from a global perspective.

## Methods

### Conceptual Approach

This observational study including demographic cross-national comparative analysis followed the relevant portions of the Strengthening the Reporting of Observational Studies in Epidemiology (STROBE) reporting guideline. This study used aggregated country-level data, and per the federal regulation 45 CFR 46 no institutional review board approval or human participant consent was sought. We started by identifying a health measure, the age-at-death distribution, which is available globally and is generated using a consistent approach. We use the frequency of deaths in 1-year increments for all countries, available through life tables (UN Population Division) from 1960 to 2021.^25^ Data were analyzed between October 2023 and January 2025. The methods for generating life tables are described elsewhere,^25,26,27^ but of importance here is that a consistent set of approaches are used to construct synthetic cohorts with age-specific mortality and age at death for all countries.

Using age at death, we began with a simple conceptual approach to health inequalities in which a state of perfect equality is one where the age at death is identical for the entire population (for example, a population where everyone dies at age 80 years, and no one dies before or after, has perfect equality). A measure of inequality, then, estimates how far away an actual distribution of death ages is from perfect equality.

We operationalized this by using the modal age at death as the age at which a country could have perfect equality. The modal age at death is the age at which the number of deaths in a life table’s synthetic cohort is the greatest. In Japan, for example, the modal age at death in 2019 was 90 years, while in India it was 78 years. We preferred this approach over using alternative approaches grounded in life expectancy because of its correspondence with the distribution of mortality (eFigure 1 in Supplement 1).

Importantly, we chose an age of perfect equality based on a country’s actual distribution of death ages to avoid fixing a priori the ideal age at death for perfect equality. Next, we estimated the variance in the distribution of age at death relative to the modal age at death. Then, we estimated the maximal possible inequality for a country’s given modal age at death. Finally, we obtained our index by dividing the actual variance by the maximal variance. The HINI shares some conceptual similarities with the Gini index of income inequality by estimating the actual distribution of age at death in the span between the ideal (perfect equality) and the worst (greatest possible inequality). We provide details of this process, and 3 examples, in eAppendix 1 in Supplement 1.

### Data Sources

Our sources of information for the distribution of ages at death are the UN Population Division life tables for all countries and territories in the World Population Prospects.^28^ The World Population Prospects provide benchmark life tables for 236 countries and territories using standardized demographic methods.^29^ Of these, we excluded small nations (mostly islands) with populations under 200 000, leaving 181 countries and territories in our main analysis.

For assessing the factors associated with health inequality, we selected a wide range of indicators from the World Bank World Development Indicators (WDI).^30^ Many WDIs have a high percentage of missingness and high degree of correlation with other WDIs. To limit investigator-based choice of factors, we first included only WDIs with more than 90% completeness for our 181 countries in 2019. Then, to reduce collinearity, we calculated the correlation between each pair of indicators and, for each pair with greater than 85% correlation, kept the indicator with less data missing. Among pairs with the same missingness, we manually selected the more general of the 2 indicators. For example, between “total fertility rate” and “total fertility rate, male” we selected total fertility rate for our final list of indicators. Using this method, we finished with 190 factors. We manually added the Gini index of income inequality given its relevance, despite a high degree of missingness, by averaging from 2010 to 2020 for a total of 191 factors.

### Additional Indices

In addition to the HINI index (eAppendix 1 in Supplement 1), we also used 2 complementary indices that capture a unidimensional measure of health inequality, can be derived from life tables, and can thus be estimated for all world countries. One index, formalized by Shkolnikov et al^21^ is by construction analogous to the Gini index for income inequality. In the health Gini, the population is ranked from fewest years lived (in practice, those who died before their first birthday) to most years lived. Then, every centile of the population is compared with the cumulative portion of person-years lived by that population. The index is defined as an area between the 45° diagonal (perfect equality of lifespan at the life expectancy) and a curve contained by the comparison divided by the whole area below the diagonal.^21^

The second index, introduced by Jamison et al,^24^ is also a unidimensional measure that uses life table information to estimate the spread of mortality. The Jamison index starts out from an assumption that a state of perfect equality takes place when the entire population lives to exactly its average life expectancy. Inequality of mortality is then calculated using the portion of all deaths in the mortality distribution that have occurred before life expectancy. For example, in a population with a life expectancy at birth of 70 years, and where 35% of deaths have taken place before the age of 70, the Jamison index value would be 1 − 0.35 = 0.65.

The HINI and these 2 indices share conceptual underpinnings in estimating health inequality as the location on the range between perfect equality (where everyone lives the exact same number of years) and the worst state of inequality. The main difference between these 2 indices and our HINI index is that they use average life expectancy as the age of perfect equality, while we prefer an approach with perfect equality at the modal age of death. The eAppendix in Supplement 1 includes details of the Gini and Jamison index construction (eAppendix 1 in Supplement 1), a figure comparing average life expectancy and modal age at death in the distribution of mortality, and the global distribution of inequality using the Gini and Jamison indices (eAppendix 2 in Supplement 1).

### Descriptive and Comparative Analysis

We used several descriptive and comparative analyses of health inequalities. We started by providing a cross-sectional description of inequalities in the year 2019 (pre–COVID-19), including breakdown by country income (are health inequalities greater in low-income vs high-income countries?) and geography. We also contextualized health inequality in the US at a global scale and over time.^31^

We identified the factors most important for health inequalities. We used a random forest analysis to identify the most important factors associated with health inequalities using all 191 indicators. The random forest classifier identifies covariate importance based on the role of each covariate in improving classification accuracy. Importance in random forests reflects a covariate’s predictive power in distinguishing between different levels of inequality not whether an association is positive or negative. Variables can achieve high importance scores through either positive or negative associations with the outcome. Random forests are well suited for handling high-dimensional data and can manage multicollinearity among variables, allowing for robust identification of importance even in the presence of correlated factors. We conducted out-of-bag error estimation using 30% of the data. Model performance was evaluated using the mean decrease in accuracy.

## Results

**Figure 1.**
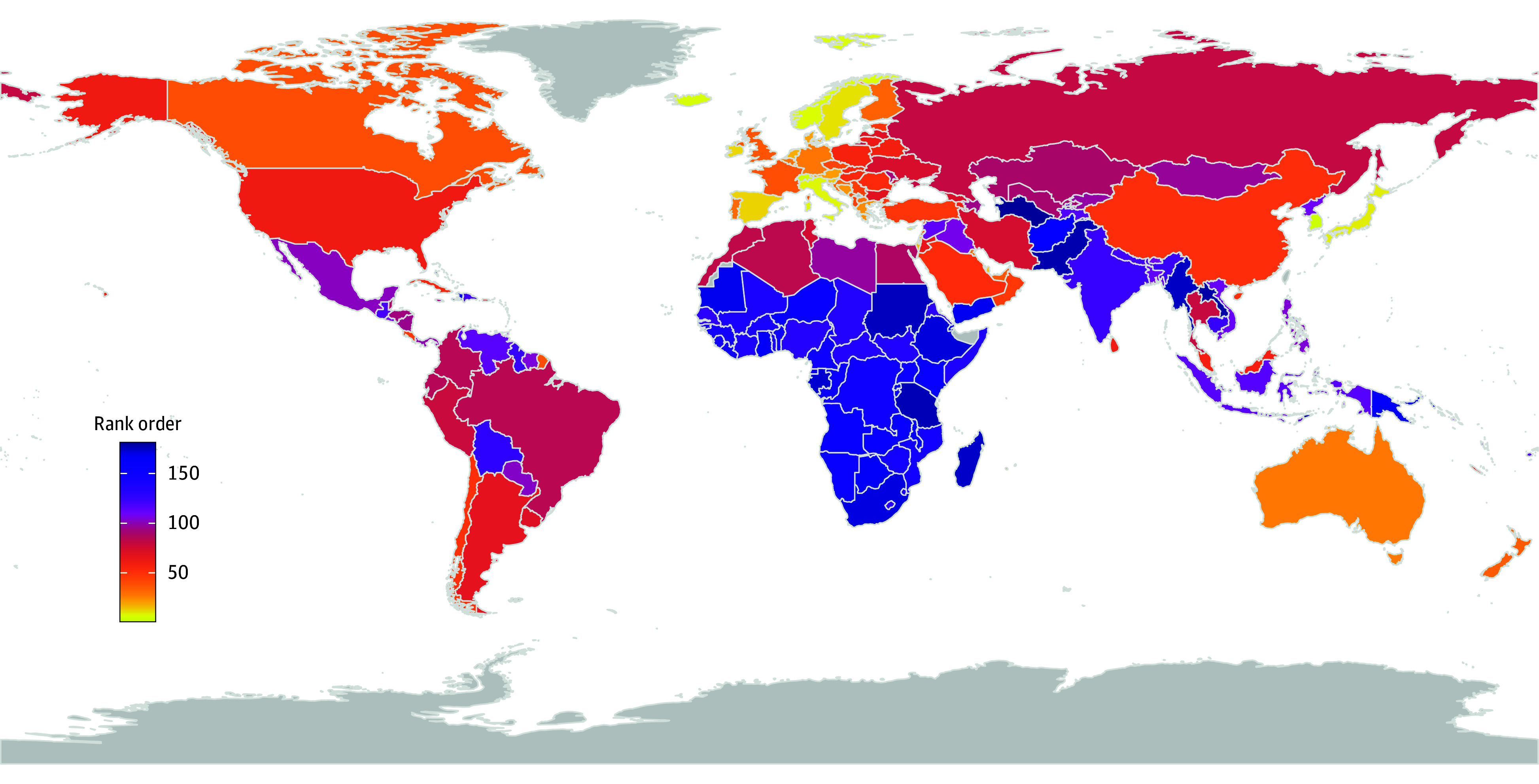
Global Distribution of Rank Order in Health Inequality Using the Health Inequality Normalized Index, 2019 The rank order from most equal (1) to most unequal of all 181 countries and territories in 2019 (pre–COVID-19). The most equal world regions (yellow and light orange) are Europe and high-income East Asia, while the most unequal regions (purple and blue) are in sub-Saharan Africa and central Asia.

Among the 181 countries and territories in 2019 (pre–COVID-19), our index ranged from 3.3 (Hong Kong, most equal) to 14.2 (Turkmenistan, most unequal), with a mean (SD) of 7.3 (3.9). The global distribution of inequality among the study’s countries (Figure 1) broadly shows that sub-Saharan African and Central Asian countries have among the world’s highest levels of inequality, while European countries have among the lowest, suggesting an association between inequality and level of population health. This suggestion is consistent in all 3 indices (eFigures 2 and 3 in Supplement 1).

In 2019, the level of inequality was highest in the 52 countries where the modal age at death (in years) was 0. Conceptually, this is a result of measuring inequality as the spread of ages at death relative to a state of perfect equality where all deaths take place at the modal age at death. Consequently, the spread of mortality relative to age 0 is greater than the spread of mortality relative to older ages in the mortality distribution.

**Figure 2.**
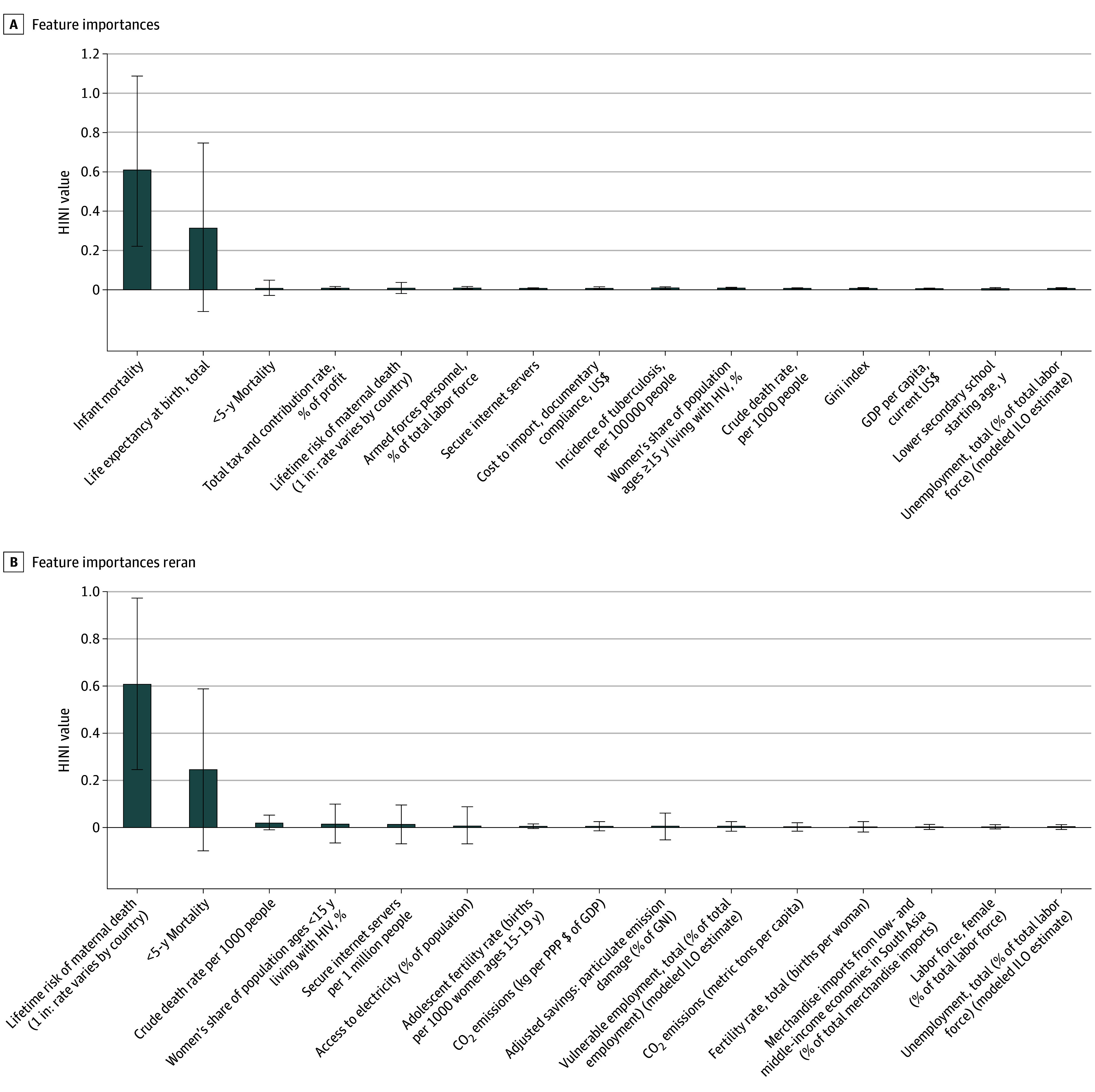
Random Forest Analysis of Indicators of Inequality A, Random forest with all 191 factors on HINI value for 181 countries and territories in 2019 shows that infant mortality and life expectancy dominate the importance scores. Among the top-15 factors in terms of importance, 7 were measures of mortality and longevity and 8 were economic (eg, GDP per capita and Gini wealth index) or social (secondary school starting age). B, Given the dominance of infant mortality and life expectancy, we reran the random forest without these factors. The top 3 factors in this analysis are measures of mortality: maternal mortality, number of children in any given year who die before reaching age 5 (under-5 mortality), and crude death rate. The random forest models with all indicators achieved an out-of-bag error rate of 5.7% on 30% of the data left out. Whiskers indicate the SD of the importance score across repeated tree shuffles. GDP indicates gross domestic product; GNI, gross national income; HINI, Health Inequality Normalized Index; ILO, International Labour Organization; PPP, purchasing power parity.

Our random forest analysis supports this observation. When predicting our inequality index using all 191 indicators, infant mortality rate and life expectancy were dominant factor importance scores, collectively accounting for more than 95% of the total factor importance (infant mortality: 65%; life expectancy: 32%). Figure 2A shows the importance of the top 15 factors. When we reran the random forest without infant mortality or life expectancy, the top 3 factors by importance were maternal mortality, number of children in any given year who die before reaching age 5 (under-5 mortality), and crude death rate, followed by factors such as HIV prevalence, electricity access prevalence, and carbon emissions (Figure 2B). Overall, the random forest models with all 191 factors achieved an out-of-bag error rate of 5.7%, indicating strong predictive accuracy.

**Figure 3.**
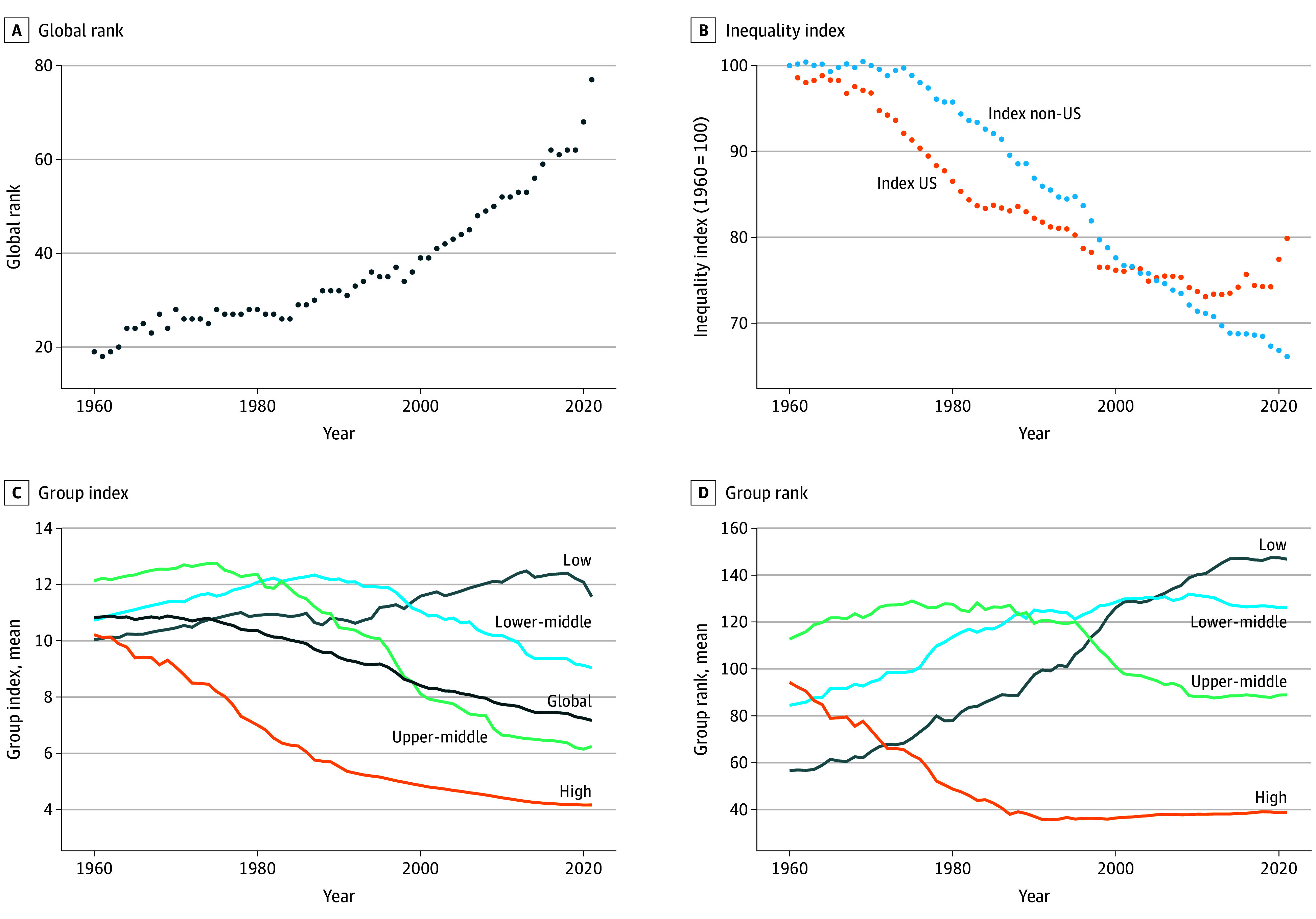
Health Inequality for the US and by World Bank Income Group The graph shows 3 trends from 1960 to 2021: A, the global rank of the US in terms of inequality increases from 19th to 77th; B, the value of the Health Inequality Normalized Index (HINI) for the US and the rest of the world. The HINI values are normalized such that 1960 values are equal to 100 to facilitate comparisons. The panel shows that while inequality in the US has decreased, the improvements were less than the rest of the world, and especially since at approximately the year 2000 when inequality improvements in the US stagnated and then reversed in 2020 and 2021. C, Index value and D, rank by World Bank Income Group from 1960 to 2021. Of note, countries were classified by the World Bank 2021 income groupings. We did not use contemporaneous income groups because they only became consistent in the 1980s and because countries that were reclassified as income thresholds changed over time. Health inequalities have decreased over time for all income groups except for low-income countries, but differences between income groups have grown such that high-income countries had higher levels of equality than all other income groups by 2021 than they were in 1960.

Health inequality in the US, in general, has decreased over time but less than many other countries, resulting in worse standing relative to other countries. Figure 3A shows that, among all world countries, the US was ranked 19th in 1960, and persistently descended in rank, so that by 2021 it was ranked 77th in the world. In percentile terms, it decreased from the 90th percentile (higher is more equal) in 1960 to the 57th in 2021. Figure 3 shows that, over the same period, the index value of inequality in the US has decreased (improved) from 1960 to 2000, then largely stagnated until COVID-19, when it reversed and increased in 2020 to 2021 to levels not seen since the mid-1990s.

When looking at trends by World Bank income group (Figure 3B and C), we observe a divergence in health inequality, with high-income countries generally experiencing reduced inequality over time (in both level and rank), and low-income countries experiencing rising inequality over time. In 1960, all 4 income groups had a similar level of inequality (index 10-12), but by 2020 the gap among income groups had grown substantially. This is consistent with our random forest findings that suggest the most important factors associated with health inequality are infant mortality and life expectancy, which have improved fastest in high-income countries and slowest in low-income countries.

## Discussion

We constructed a novel index of health inequality that can be estimated using age at death from life tables for all countries and over time. By analyzing patterns and correlates of health inequality, we identified 3 key patterns. First, the principal drivers of health inequality are infant mortality and life expectancy. Second, average global health inequalities have decreased over time, although this overall pattern belies increasing inequality in low-income countries and divergence in inequality since 1960. Finally, the pattern in the US shows stagnating improvement in health inequity since the early 2000s, increasing inequities during the COVID-19 years, and decreasing health inequality compared with other high-income countries. Our global perspective can help countries contextualize their levels of health inequality as large or small.

Infant mortality—and life expectancy by extension—are dominant drivers of health inequalities. The most dramatic declines in health inequalities have taken place in those countries that escaped having the first year of life be the most dangerous year, and the greatest gains in future health inequalities stand to come from reducing infant mortality in those countries where infancy remains the modal age at death. As of 2021, 50 countries in which the modal age at death was zero years remain. Viewed this way, high infant mortality is a proxy for high levels of inequality, and driving infant mortality down in those 50 countries may be the most effective - and feasible - approach for reducing inequality. Among countries with lower infant mortality, mortality among young children remains an important factor driving health inequality, because of the role mortality during early life plays in stretching the distribution of mortality away from equality.

The global decrease in average health inequality between 1960 and 2021 partly reflects global progress in infant mortality and life expectancy but also demonstrates a substantial divergence. Health inequalities in the 59 high-income countries have declined dramatically over the study period. In the 93 middle-income countries, health inequalities largely stagnated until the 1980s, and have been declining since. Finally, in the 29 low-income countries, health inequalities have steadily increased over the study period, partly reflecting an intermediate stage of inequality in which small improvements in infant mortality increase inequality so long as infant mortality dominates the mortality distribution. This trend will reverse when infant mortality stops being the most common age at death in those countries.

The trend in health inequality in the US in absolute terms as well as relative to comparison countries is stark. Health inequality in the US has declined since 1960, but less relative to other high-income countries. In 1960, the US was ranked 19th in the world among 181 world countries and territories, but by 2021 it was ranked 77th. In 2021, the US had the 5th worst level of health inequities among 59 high income countries, below Lithuania, Uruguay, and Brunei. The relative position of health inequities in the US decreased meaningfully during 2020 and 2021. This is consistent with the observation that the age distribution of COVID-19 mortality was less concentrated among the elderly in the US as in other countries, increasing the national spread (inequality) of age at death.

### Strengths and Limitations

Our analysis uses a newly constructed index of health inequality, so its properties deserve discussion. First, the measure of health it uses is all-cause mortality. This is both a measure of broad relevance and a narrow lens to examine inequality insofar as it may not represent other dimensions of health. This index could be constructed for other measures of health, and may provide additional insights, as long as they are comparable across countries and over time. Second, the assumption of perfect equality as lifespan for all equal to the modal age at death is both common and unusual. Most measures of inequality explicitly or implicitly assume a state of perfect equality as the benchmark against which to compare actual variation. However, since the HINI index takes perfect equality to be equal lifespans at the modal age at death, we construct the index using perfect equality for which, in countries with high infant mortality, the entire population dies in the first year of life. In this sense, the HINI is a value-free measure of health inequality. The main implication of this construction is that the transition of a country from a state where the modal age at death is 0 to 1 where it is some older age entails a discontinuous improvement in inequality. Third, we relied on life table data from the UN Population Division, which in turn relies on multiple data sources and statistical models to estimate the complete life table distributions. To the extent that precise age-specific mortality rates may be measured with error, this may impact our HINI index value although not the overall study themes. Finally, we used factors collected by the World Bank to assess correlates of inequality. Several of the important factors, especially those reflecting early life mortality, may be correlated. This limits the extent to which these reflect independent factors, and further highlights the importance of infant mortality as a basis for inequality. These indicators are also not causal, and in addition may be missing important factors that are not collected by the World Bank.

## Conclusions

Results of this observational study including demographic cross-national comparative analysis suggest that reducing infant mortality and extending life expectancy are not only desirable and achievable goals for their own sake but are also a direct way to reduce health inequalities globally.

## Notes

Supplement 1. **eAppendix 1.** Construction of Inequality Indices **eFigure 1.** Distribution of age at death in a synthetic cohort of 100,000 people in India and the US, from 2019 life tables **eAppendix 2.** Spatial Distribution of Gini and Jamison Indices **eFigure 2.** Global distribution of rank order in health inequality using Gini, 2019 **eFigure 3.** Global distribution of rank order in health inequality using Jamison Index, 2019 **eAppendix 3.** 2019 HINI values, country income group, and covariates

Supplement 2.
Data Sharing Statement
