# Supplementary material for: A Global Analysis of Within-Country Health Inequalities
Source: JAMA Health Forum. 2025 Oct 17;6(10):e253611. doi: 10.1001/jamahealthforum.2025.3611 (PMC12534847; doi:10.1001/jamahealthforum.2025.3611)
Supplement: Supplement 1. — eAppendix 1. Construction of Inequality Indices eFigure 1. Distribution of age at death in a synthetic cohort of 100,000 people in India and the US, from 2019 life tables eAppendix 2. Spatial Distribution of Gini and Jamison Indices eFigure 2. Global distribution of rank order in health inequality using Gini, 2019 eFigure 3. Global distribution of rank order in health inequality using Jamison Index, 2019 eAppendix 3. 2019 HINI values, country income group, and covariates [file jamahealthforum-e253611-s001.pdf]

Supplemental Online Content

Bendavid E, Littleton AKS, Mejía-Guevara I, Fenyo M, Miller G. A global analysis of within-country health inequalities. *JAMA Health Forum.* 2025;6(10):e253611. doi:10.1001/jamahealthforum.2025.3611

eAppendix 1. Construction of Inequality Indices

eFigure 1. Distribution of age at death in a synthetic cohort of 100,000 people in India and the US, from 2019 life tables

eAppendix 2. Spatial Distribution of Gini and Jamison Indices

eFigure 2. Global distribution of rank order in health inequality using Gini, 2019

eFigure 3. Global distribution of rank order in health inequality using Jamison Index, 2019

eAppendix 3. 2019 HINI values, country income group, and covariates

This supplemental material has been provided by the authors to give readers additional information about their work.

© 2025 Bendavid E et al. *JAMA Health Forum*.

SUPPLEMENTARY MATERIALS

Supplementary eAppendix 1 – Construction of Inequality Indices

We describe the step-by-step construction of the principal index used in this analysis, HINI. In addition, we describe the construction of 2 additional indices that we used as a form of robustness analysis since they enable some of the principal comparisons we perform with the HINI. We call these two indices the Jamison index, after the authors describing the index, and the Gini health inequality index, which is based on the conceptual foundations of the Gini index of income inequality.

Health Inequality Normalized Index (HINI)

We created a new unidimensional inequality index to measure inequality in age at death in each country based on standard life tables. We start by taking the modal age at death for each country, which is the 1-year interval with the largest number of deaths from a life table. Since all ages at and above 100 are lumped in life tables from the United Nations, many countries have an apparent modal age of 100+. In these cases, we use the 1-year interval with the second most number of deaths as our modal value (in a sensitivity analysis we extrapolated the 100+ group up to age 110, as a linear extension of mortality in the age interval 90-99. That yielded an identical choice of modal age at death as selecting the second most common age interval).

Next, we calculated the maximal (hypothetical) inequality in age at death for each country, given its modal age at death. The conceptual foundation for the maximal

© 2025 Bendavid E et al. *JAMA Health Forum*.

inequality is that maximal *equality* of age at death is when everyone in a country dies at the same age. The variance of such a distribution is 0 (e.g. if everyone in the country died at age 82). By extension, the most *unequal* distribution for the same country has the greatest possible variance in age at death, while respecting the modal age at death. This was operationalized by redistributing age at death such that 2% of the population dies at the observed mode, and the rest of the deaths are evenly distributed at ~1% each among the remaining 100 non-modal ages from 0 to 100+. For example, we find the modal age at death in the US to be 87. Then, we calculate the maximal possible variance for the US by assuming that 2% of deaths took place at age 87, and the other 98% of deaths are distributed evenly among the remaining 100 possible ages-at-death (0.98% at age 0, 1, 2…100+, excluding 87).

The variance representing the maximum inequality is the mode-variance of this distribution: $Variance_{maximal} = \sum_{i=0, i \neq mode}^{i=100} [(age_i - mode)^2 \times 0.98]$.

After calculating the maximum possible inequality as the variance of the most unequal distribution in age at death, we calculate the variance of the actual distribution as: $Variance_{actual} \sum_{i=0, i \neq mode}^{i=100} [(age_i - mode)^2 \times w_i]$ where w_i is the actual portion of deaths at age i. The index of inequality is the ratio of these two estimates (scaled up by 10^3 for interpretability), $Index = \frac{Variance_{actual}}{Variance_{maximal}} x\ 10^3$. This way the Index measures the actual dispersion in age at death, relative to the maximal possible dispersion, standardized for the modal age at death in the country. The Index is constructed such that higher values imply greater inequality (the actual variance is closer to the maximal variance for the country).

© 2025 Bendavid E et al. *JAMA Health Forum*.

For the United States in 2019, for example, the modal age at death was 87. Given that, the $Variance_{maximal}$ is 3899.2. The $Variance_{actual}$ for the US in that year was 18.8, coming to a HINI value of 4.8. Another example is Nigeria, which, in 2019, had infant mortality high enough that the modal age at death was 0. Given that, we estimate that the $Variance_{maximal}$ was 5024.4, while the $Variance_{actual}$ was 59.5, resulting in a HINI value of 11.8.

Jamison Index

We constructed the Jamison index using the original methodology, which uses survival curves to conceptualize inequality.[23] Perfect equality is defined as a square survival curve, where everyone dies at the same age (the life expectancy is used by definition). A country's inequality is then measured by the rectangularity of its survival curve – i.e. the area above its actual survival curve and below the model rectangular survival curve, or A in the figure below (reproduced from the original article):

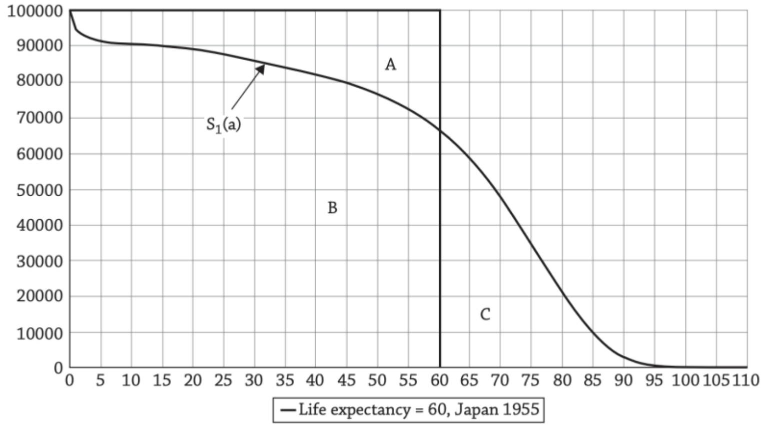

© 2025 Bendavid E et al. *JAMA Health Forum*.

The mathematical rectangularity definition is as follows, with e_s denoting life expectancy:

$$R_s = (A/e_s)$$

Based on this definition, we created survival curves from the life table data and using numerical integration by single age to estimate A, calculated Jamison indices for all country-years.

Gini

We computed the relative Gini index, following Hanada and Shkolnikov.[21,31] The index is estimated as:

$$G_0 = (1/e_0) * [e_0 - (1/l(0)^2)\int^{\omega} l(x)^2 dx \ ] = {G_0}^{abs}/e_0,$$

where ω is the highest age in the population, l(x) is the survival function, e_0 represents the life expectancy at birth, and *l(0)* is the life table radix (=100000). The absolute Gini coefficient (${G_0}^{abs}$) measures the average difference, in years, between the ages at death of any pair of individuals. To compare populations with varying life expectancy levels, we divide the Gini index by the life expectancy, yielding the relative Gini coefficient (G_0). This dimensionless indicator represents variation as a proportion of life expectancy, which is unaffected by proportional changes across all lifespans in a population, even if the absolute difference in years changes.

A Note on Index Choice

We note above that we used the HINI index as the default measure because of its intuitive relationship to the distribution of mortality. This is demonstrated below in the figure

© 2025 Bendavid E et al. *JAMA Health Forum*.

below of the number of deaths at each age (for India and the US in 2019). The figure shows the modal age at death (dashed line) as corresponding to the peak mortality, while life expectancy (dotted line) does not have similarly intuitive correspondence.

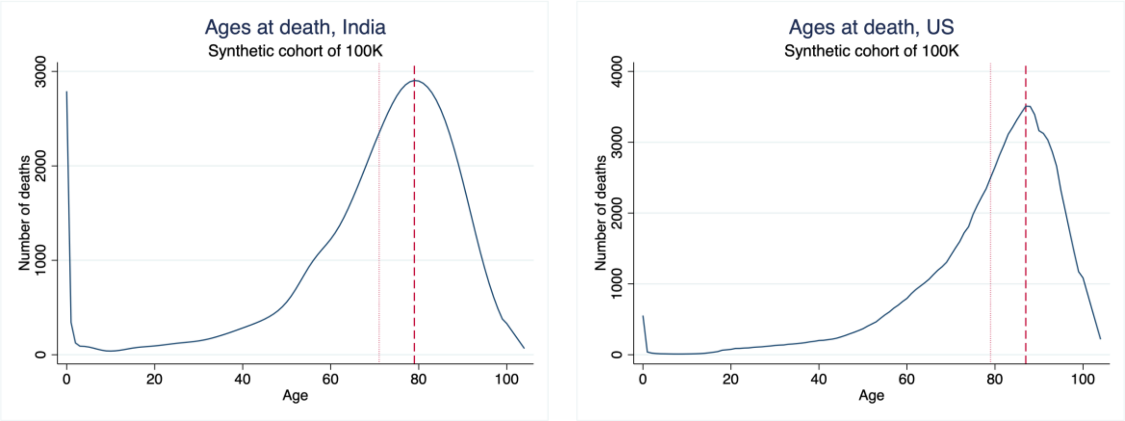

Supplementary eFigure 1: distribution of age at death in a synthetic cohort of 100,000 people in India and the US, from 2019 life tables. The figures show the modal age at death and the life expectancy for each country.

© 2025 Bendavid E et al. *JAMA Health Forum*.

Supplementary eAppendix 2: Spatial Distribution of Gini and Jamison Indices

Supplementary eFigure 2: global distribution of rank order in health inequality using Gini, 2019

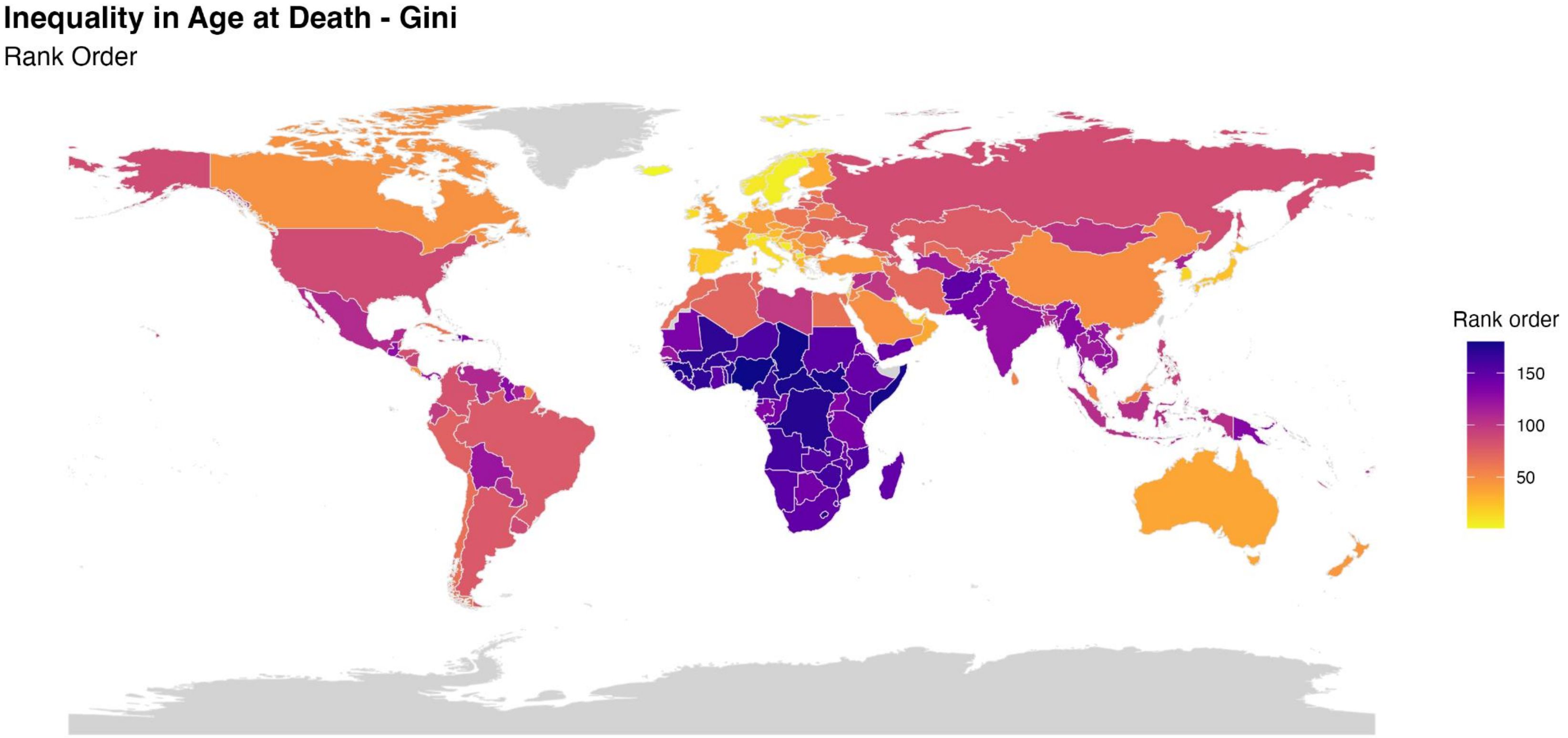

Supplementary eFigure 3: global distribution of rank order in health inequality using Jamison Index, 2019

© 2025 Bendavid E et al. *JAMA Health Forum*.

Inequality in Age at Death - Jamison

Rank Order

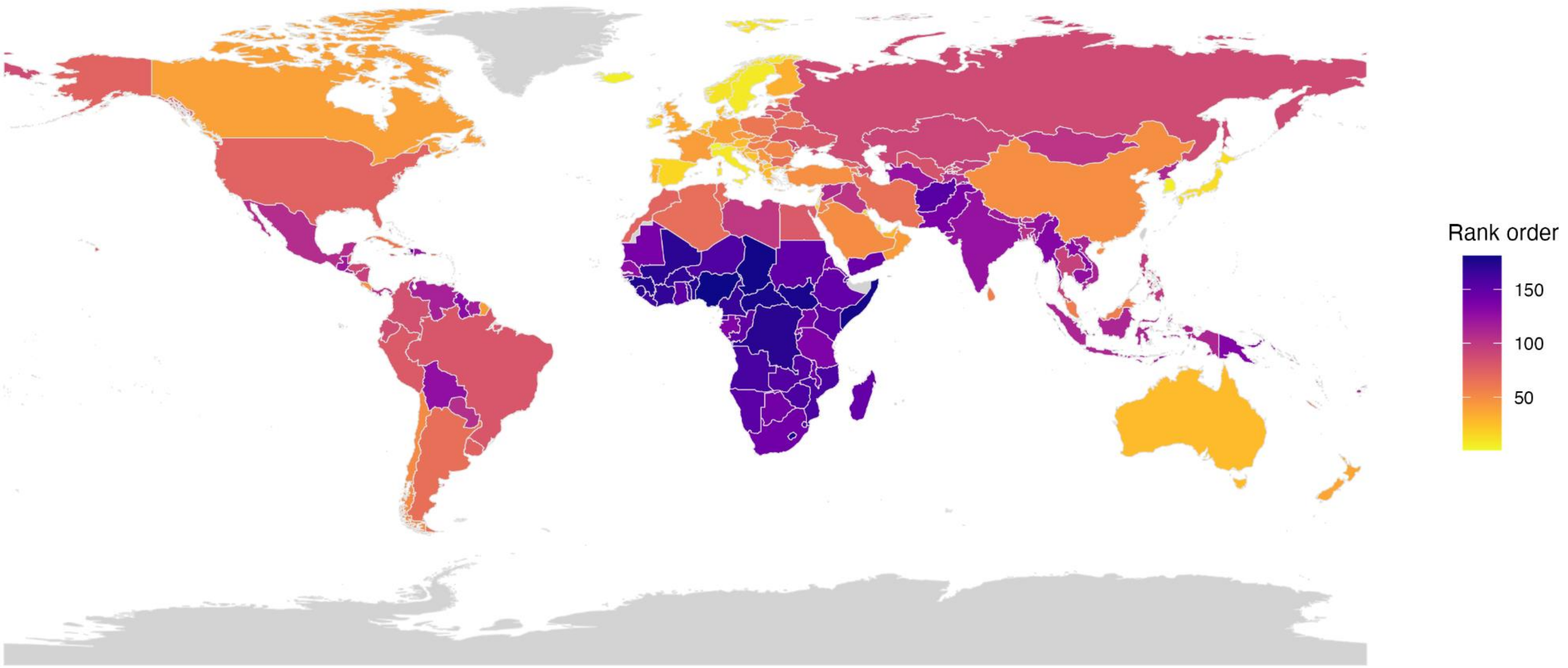

© 2025 Bendavid E et al. *JAMA Health Forum*.

Supplementary eAppendix 3: 2019 HINI values, country income group, and covariates

HINI – Health Inequality Normalized Index
WB Income – 1: low-income countries; 2: low-middle income; 3: high-middle income; 4: high-income countries
Modal AAD – modal age-at-death
U5MR – under-5 mortality rate (per 1,000 births)
GDPpc – gross domestic product per capita

Country	HINI	WB Income	Modal AAD	U5MR	GDPpc	Pop	Fert. Rate	Corruption	Gov. Effectivns	Gini	[illegible]
China, Hong Kong SAR	3.33	4	90	2.02	59.59	7.51	1.1	1.7	1.7		[illegible]
China, Macao SAR	3.36	4	90	3.39	127.27	0.66	1.1	0.7	1.2		[illegible]
Singapore	3.48	4	89	2.40	98.41	5.70	1.1	2.2	2.2		[illegible]
Republic of Korea	3.49	4	89	3.14	42.76	51.76	0.9	0.7	1.4	31.7	[illegible]
Switzerland	3.52	4	89	3.77	70.94	8.58	1.5	2.0	1.9	32.8	[illegible]
Iceland	3.54	4	88	1.78	56.94	0.36	1.7	1.7	1.5	27.6	[illegible]
Norway	3.58	4	88	2.55	64.39	5.35	1.5	2.1	1.9	26.8	[illegible]
Italy	3.59	4	90	2.88	42.71	59.73	1.3	0.3	0.5	34.7	[illegible]
Japan	3.59	4	90	2.57	42.02	126.63	1.4	1.5	1.6	33.3	[illegible]
Cyprus	3.61	4	87	2.82	41.52	1.23	1.3	0.6	1.0	32.9	[illegible]
Sweden	3.63	4	90	2.50	52.85	10.28	1.7	2.1	1.7	28.4	[illegible]
Ireland	3.64	4	87	3.03	86.65	4.93	1.7	1.5	1.3	32.2	[illegible]
Spain	3.64	4	90	3.13	40.80	47.13	1.2	0.7	1.0	35.2	[illegible]
Slovenia	3.65	4	88	2.56	38.95	2.09	1.6	0.9	1.1	24.9	[illegible]
Qatar	3.67	4	86	6.31	89.97	2.81	1.8	0.8	0.7		[illegible]
French Polynesia	3.69	4	87	6.82		0.30	1.7				[illegible]
Israel	3.70	4	89	3.99	40.63	9.05	3.0	0.8	1.3	40.5	[illegible]
Netherlands	3.72	4	88	4.21	56.78	17.34	1.6	1.9	1.8	28.4	[illegible]
Malta	3.74	4	89	7.28	43.95	0.50	1.1	0.2	0.9	29.3	[illegible]
Denmark	3.81	4	88	3.38	57.16	5.81	1.7	2.2	1.9	27.6	[illegible]
Austria	3.82	4	88	3.73	55.83	8.88	1.5	1.6	1.5	30.6	[illegible]
Maldives	3.84	3	85	7.01	19.51	0.50	1.7	-0.3	-0.1	33.0	[illegible]
Bosnia and Herzegovina	3.86	3	79	5.87	14.90	3.36	1.4	-0.6	-0.7	33.1	[illegible]
Greece	3.86	4	88	4.16	29.70	10.72	1.3	0.0	0.3	34.6	[illegible]
Luxembourg	3.86	4	89	5.04	116.52	0.62	1.3	2.1	1.7	32.6	[illegible]
Bahrain	3.88	4	85	7.10	45.31	1.49	1.9	0.0	0.3		[illegible]
Australia	3.89	4	90	3.55	49.31	25.34	1.7	1.8	1.6	34.5	[illegible]
Germany	3.89	4	84	3.77	53.93	83.09	1.5	1.9	1.5	31.1	[illegible]
Belgium	3.90	4	88	4.15	51.94	11.49	1.6	1.5	1.1	27.9	[illegible]
North Macedonia	3.93	3	81	7.20	16.77	2.08	1.3	-0.4	-0.1	36.9	[illegible]
Portugal	3.93	4	89	3.53	34.95	10.29	1.4	0.8	1.2	35.3	[illegible]
Kuwait	3.94	4	85	8.98	49.85	4.44	2.2	-0.1	0.0		[illegible]
Finland	3.94	4	89	2.48	48.58	5.52	1.4	2.2	2.0	27.4	[illegible]
Croatia	3.97	4	85	4.61	29.34	4.07	1.5	0.1	0.5	31.4	[illegible]
New Zealand	3.99	4	88	4.36	42.88	4.98	1.7	2.2	1.7		[illegible]

© 2025 Bendavid E et al. *JAMA Health Forum*.

United Arab Emirates	3.99	4	85	6.93	68.26	9.21	1.3	1.1	1.4	29.3	[illegible]
United Kingdom	3.99	4	88	4.27	47.37	66.84	1.6	1.8	1.5	34.0	[illegible]
France	4.03	4	90	4.37	46.02	67.39	1.9	1.3	1.4	32.6	[illegible]
Canada	4.11	4	90	5.01	49.17	37.60	1.5	1.8	1.7	33.5	[illegible]
Montenegro	4.11	3	82	3.15	21.53	0.62	1.8	0.0	0.1	38.6	[illegible]
Lebanon	4.11	3	84	7.23	14.56	5.78	2.1	-1.2	-0.8	31.8	[illegible]
Czechia	4.15	4	87	3.08	40.98	10.67	1.7	0.6	0.9	25.9	[illegible]
Albania	4.19	3	86	9.80	13.66	2.85	1.4	-0.5	0.0	31.8	[illegible]
Serbia	4.21	3	82	5.78	18.31	6.95	1.5	-0.4	0.0	38.1	[illegible]
Oman	4.35	4	84	11.32	31.28	4.60	2.8	0.4	0.3		[illegible]
Costa Rica	4.35	3	87	8.19	20.94	5.08	1.6	0.7	0.4	48.7	[illegible]
Türkiye	4.37	3	83	10.09	28.20	83.48	2.0	-0.3	0.0	40.5	[illegible]
Estonia	4.38	4	86	2.16	36.40	1.33	1.7	1.6	1.2	32.1	[illegible]
Chile	4.48	4	86	7.45	24.97	19.04	1.5	1.0	0.9	45.4	[illegible]
China	4.52	3	84	7.93	15.98	1407.75	1.5	-0.3	0.6	40.3	[illegible]
Saudi Arabia	4.53	4	84	7.56	46.96	35.83	2.5	0.3	0.3		[illegible]
Romania	4.56	4	84	7.19	29.88	19.37	1.8			35.9	[illegible]
Hungary	4.58	4	85	4.37	32.55	9.77	1.6	0.1	0.5	29.6	[illegible]
Slovakia	4.58	4	86	6.10	31.93	5.45	1.6	0.2	0.6	25.8	[illegible]
Cuba	4.63	3	85	5.22		11.32	1.6	0.0	-0.2		[illegible]
Poland	4.64	4	87	4.38	33.19	37.97	1.4	0.6	0.5	32.4	[illegible]
Belarus	4.66	3	80	3.13	19.28	9.42	1.4	0.0	-0.1	26.6	[illegible]
Malaysia	4.71	3	82	8.58	28.42	32.80	1.8	0.3	1.0	43.0	[illegible]
Sri Lanka	4.73	2	83	7.32	13.07	21.80	2.0	-0.3	-0.1	38.0	[illegible]
Armenia	4.78	3	81	11.52	13.65	2.82	1.6	-0.2	-0.2	30.5	[illegible]
United States of America	4.82	4	87	6.47	62.63	328.33	1.7	1.2	1.5	41.0	[illegible]
Lithuania	4.85	4	85	4.01	37.17	2.79	1.6	0.7	1.0	35.8	[illegible]
New Caledonia	4.85	4	87	12.89		0.27	2.1				[illegible]
Puerto Rico	4.86	4	87	10.43	34.80	3.19	1.0	0.0	-0.2		[illegible]
Jordan	4.87	3	82	15.29	10.07	10.70	2.9	0.1	0.1	33.2	[illegible]
Barbados	4.87	4	83	12.61	15.64	0.28	1.6	1.2	0.6		[illegible]
Argentina	4.88	3	83	11.37	22.07	44.94	2.0	-0.1	-0.1	42.6	[illegible]
Bulgaria	4.90	3	84	6.67	23.27	6.98	1.6	-0.2	0.2	37.3	[illegible]
Latvia	4.91	4	84	4.08	31.01	1.91	1.6	0.5	1.1	35.5	[illegible]
Uruguay	4.92	4	83	6.63	23.03	3.43	1.5	1.2	0.7	41.7	[illegible]
Brunei Darussalam	5.05	4	82	11.03	62.10	0.44	1.8	0.8	1.3		[illegible]
Georgia	5.05	3	81	9.51	14.99	3.72	2.0	0.7	0.8	37.6	[illegible]
Ukraine	5.10	2	84	7.49	12.80	44.39	1.2	-0.8	-0.3	25.5	[illegible]
Iran (Islamic Republic of)	5.13	3	84	13.49	12.39	86.56	1.8	-1.1	-0.6	40.2	[illegible]
Tunisia	5.15	2	83	16.87	11.42	12.05	2.1	-0.1	0.0	34.3	[illegible]
Mauritius	5.22	4	83	15.97	22.87	1.27	1.4	0.2	0.9	37.7	[illegible]
Cabo Verde	5.22	2	83	14.91	7.17	0.58	1.9	0.9	0.3	44.8	[illegible]
Peru	5.24	3	85	13.34	12.85	32.82	2.2	-0.5	-0.1	44.6	[illegible]
Thailand	5.24	3	87	9.05	18.45	71.31	1.4	-0.5	0.3	37.6	[illegible]
Russian Federation	5.25	3	84	5.49	27.21	144.41	1.5	-0.8	0.1	39.1	[illegible]
Morocco	5.28	2	82	19.31	7.55	36.30	2.4	-0.3	-0.2	39.5	[illegible]
Algeria	5.30	2	84	23.26	11.52	42.71	3.0	-0.6	-0.5	27.6	[illegible]

© 2025 Bendavid E et al. *JAMA Health Forum*.

Brazil	5.32	3	85	14.98	14.76	211.78	1.7	-0.4	-0.2	53.0	7
Colombia	5.33	3	86	13.66	14.59	50.19	1.8	-0.3	0.1	52.5	7
Ecuador	5.34	3	86	13.44	11.37	17.34	2.1	-0.5	-0.3	47.0	7
Belize	5.35	3	82	12.27	7.25	0.39	2.1	-0.2	-0.6		7
Egypt	5.38	2	78	20.18	11.76	105.62	3.0	-0.6	-0.2	30.6	7
Kazakhstan	5.38	3	79	10.16	26.35	18.51	2.9	-0.3	0.1	27.9	7
Uzbekistan	5.40	2	79	14.87	7.35	33.58	2.8	-1.0	-0.5		7
Republic of Moldova	5.41	2	77	14.74	13.03	2.66	1.8	-0.6	-0.4	29.2	7
Jamaica	5.49	3	80	14.20	9.78	2.81	1.4	-0.1	0.6		7
Honduras	5.51	2	80	17.01	5.74	9.96	2.4	-0.9	-0.6	51.3	7
Azerbaijan	5.54	3	80	20.46	14.44	10.02	1.8	-0.8	-0.1		7
Nicaragua	5.66	2	85	16.63	5.45	6.66	2.4	-1.0	-0.7	45.1	7
Mongolia	5.68	2	79	16.12	12.49	3.23	2.9	-0.4	-0.2	33.4	7
Libya	5.69	3	81	12.96	15.17	6.57	2.5	-1.6	-1.8		7
Kyrgyzstan	5.69	2	79	18.32	5.26	6.46	3.3	-0.9	-0.7	29.0	7
Panama	5.72	4	87	17.94	31.44	4.23	2.4	-0.6	0.1	51.1	7
Samoa	5.77	3	80	19.30	6.63	0.21	4.0	0.7	0.5	40.4	7
Mexico	5.77	3	85	14.14	19.68	125.09	1.9	-0.9	-0.3	47.8	7
Paraguay	5.81	3	81	19.61	12.62	6.53	2.5	-0.9	-0.6	48.7	7
Trinidad and Tobago	5.85	4	84	17.73	25.83	1.52	1.6	-0.2	0.1		7
Philippines	5.93	2	80	27.15	8.91	110.38	2.8	-0.6	0.1	44.9	7
Suriname	5.98	3	81	18.10	19.04	0.60	2.4	-0.4	-0.6		7
Solomon Islands	5.99	2	79	20.05	2.66	0.67	4.1	0.0	-1.0	37.1	7
Iraq	5.99	3	80	26.49	10.94	41.56	3.6	-1.4	-1.3	29.5	7
El Salvador	6.01	2	82	13.33	8.78	6.28	1.8	-0.5	-0.5	42.0	7
Dem. People's Republic of Korea	6.02	1	83	17.30		25.76	1.8	-1.6	-1.4		7
Bhutan	6.03	2	80	28.39	11.86	0.77	1.5	1.6	0.4	38.1	7
Viet Nam	6.05	2	83	21.28	8.04	95.78	1.9	-0.5	0.0	36.1	7
Bangladesh	6.06	2	81	30.71	4.75	165.52	2.0	-1.0	-0.7	32.3	7
Syrian Arab Republic	6.07	1	81	22.24		20.10	2.8	-1.7	-1.7		7
Venezuela (Bolivarian Republic of)	6.08	3	84	18.65		28.97	2.3	-1.5	-1.7		7
Indonesia	6.14	3	79	23.89	11.81	269.58	2.2	-0.5	0.2	38.6	7
Tajikistan	6.17	1	79	33.33	3.58	9.34	3.3	-1.3	-1.1	32.3	7
Fiji	6.20	3	75	26.91	13.68	0.92	2.5	0.7	0.8	35.9	6
Vanuatu	6.23	2	78	25.71	3.14	0.30	3.8	-0.3	-0.5	34.9	6
Guatemala	6.36	3	83	24.49	8.65	16.60	2.6	-0.9	-0.7	48.3	7
Nepal	6.43	2	79	29.44	3.95	28.83	2.1	-0.7	-1.1	32.8	6
Sao Tome and Principe	6.44	2	79	16.08	4.01	0.21	4.0	0.2	-0.7	35.8	6
Dominican Republic	6.49	3	84	34.34	18.41	10.88	2.3	-0.8	-0.3	45.5	7
India	6.61	2	79	34.37	6.71	1383.11	2.1	-0.3	0.2	35.3	7
Cambodia	6.63	2	81	26.67	4.39	16.21	2.4	-1.3	-0.6		7
Guyana	6.77	3	76	29.28	13.08	0.80	2.4	-0.1	-0.4		6
Bahamas	6.84	4	84	23.49	36.43	0.40	1.4	1.1	0.5		7

© 2025 Bendavid E et al. *JAMA Health Forum*.

Bolivia (Plurinational State of)	6.92	2	77	37.87	8.72	11.78	2.7	-0.8	-0.8	46.7	[illegible]
Senegal	6.97	2	79	39.80	3.36	16.00	4.5	0.0	-0.1	39.2	[illegible]
Eritrea	7.64	1	80	40.55		3.50	4.0	-1.4	-1.8		[illegible]
Lesotho	11.80	2	0	90.59	2.58	2.23	3.1	-0.1	-0.9	44.9	[illegible]
Nigeria	11.84	2	0	117.03	5.14	203.30	5.4	-1.1	-1.2	35.6	[illegible]
Chad	11.89	1	0	113.61	1.58	16.13	6.4	-1.4	-1.6	40.4	[illegible]
Central African Republic	12.08	1	0	106.71	0.95	5.21	6.0	-1.2	-1.8	56.2	[illegible]
South Sudan	12.31	1	0	99.08		10.45	4.6	-1.8	-2.4	45.2	[illegible]
Somalia	12.60	1	0	118.90	1.19	15.98	6.5	-1.7	-2.3	36.8	[illegible]
Côte d'Ivoire	12.73	2	0	80.13	5.21	26.15	4.5	-0.5	-0.5	40.6	[illegible]
Eswatini	12.81	2	0	49.28	8.65	1.17	2.9	-0.3	-0.6	53.1	[illegible]
Mali	12.87	1	0	94.70	2.32	20.57	6.1	-0.7	-1.1	34.6	[illegible]
Burkina Faso	12.91	1	0	88.58	2.18	20.95	5.0	-0.2	-0.8	40.8	[illegible]
Togo	12.97	1	0	66.50	2.12	8.24	4.4	-0.7	-0.9	43.8	[illegible]
Zimbabwe	12.97	2	0	53.12	3.63	15.35	3.6	-1.3	-1.3	45.9	[illegible]
Guinea	12.98	1	0	98.21	2.57	12.88	4.6	-0.9	-0.8	34.2	[illegible]
Mozambique	12.99	1	0	72.97	1.28	30.29	4.8	-0.9	-0.9	49.8	[illegible]
Sierra Leone	13.01	1	0	107.19	1.71	8.05	4.2	-0.4	-1.1	34.9	[illegible]
Guinea-Bissau	13.01	1	0	79.51	1.94	1.97	4.2	-1.5	-1.6	42.8	[illegible]
Democratic Republic of the Congo	13.02	1	0	84.50	1.10	89.91	6.3			42.1	[illegible]
Liberia	13.03	1	0	80.31	1.47	4.99	4.3	-0.9	-1.4	35.0	[illegible]
Benin	13.07	2	0	88.54	3.29	12.29	5.1	-0.3	-0.5	43.0	[illegible]
Cameroon	13.13	2	0	74.76	3.74	25.78	4.6	-1.2	-0.8	44.7	[illegible]
Uganda	13.14	1	0	44.67	2.18	42.95	4.8	-1.1	-0.6	42.7	[illegible]
Equatorial Guinea	13.14	3	0	80.85	18.50	1.55	4.4	-1.5	-1.0		[illegible]
Congo	13.19	2	0	51.62	3.83	5.57	4.3	-1.4	-1.4	48.9	[illegible]
Kenya	13.23	2	0	42.75	4.45	50.95	3.5	-0.8	-0.4	40.8	[illegible]
Namibia	13.25	3	0	41.12	9.81	2.45	3.4	0.3	0.1	60.1	[illegible]
Burundi	13.26	1	0	56.76	0.75	11.87	5.3	-1.5	-1.4	38.6	[illegible]
Zambia	13.27	2	0	64.03	3.47	18.38	4.5	-0.7	-0.7	56.4	[illegible]
Angola	13.28	2	0	74.15	6.67	32.35	5.4	-1.1	-1.1	47.0	[illegible]
Niger	13.37	1	0	80.67	1.22	23.44	7.0	-0.5	-0.8	35.1	[illegible]
Djibouti	13.39	2	0	58.04	5.53	1.07	2.9	-0.9	-0.8	43.6	[illegible]
Afghanistan	13.41	1	0	60.50	2.07	37.77	4.9	-1.4	-1.5		[illegible]
Malawi	13.42	1	0	41.23	1.54	18.87	4.1	-0.8	-0.8	42.9	[illegible]
Comoros	13.46	2	0	62.42	3.06	0.79	4.1	-1.0	-1.7	45.3	[illegible]
Gambia	13.48	1	0	51.25	2.22	2.51	4.9	-0.4	-0.7	39.8	[illegible]
Haiti	13.52	1	0	62.43	3.07	11.16	2.9	-1.3	-2.0	41.1	[illegible]
Papua New Guinea	13.62	2	0	44.97	4.29	9.54	3.3	-1.0	-0.8	41.9	[illegible]
Ghana	13.63	2	0	46.39	5.54	31.52	3.7	-0.1	-0.3	43.0	[illegible]
Botswana	13.68	3	0	46.50	16.35	2.50	2.9	0.7	0.4	56.9	[illegible]
Yemen	13.68	1	0	61.58		31.55	4.0	-1.7	-2.3	36.7	[illegible]
Mauritania	13.76	2	0	64.90	5.34	4.38	4.5	-0.8	-0.6	34.2	[illegible]
Rwanda	13.80	1	0	41.73	2.23	12.84	3.9	0.5	0.1	45.3	[illegible]
South Africa	13.82	3	0	35.23	13.71	58.09	2.5	0.0	0.2	63.1	[illegible]

© 2025 Bendavid E et al. *JAMA Health Forum*.

Ethiopia	13.84	1	0	50.84	2.22	114.12	4.3	-0.4	-0.6	34.1	[illegible]
Gabon	13.84	3	0	41.74	14.95	2.24	3.6	-0.9	-0.9	38.0	[illegible]
Madagascar	13.85	1	0	51.92	1.62	27.53	4.0	-1.0	-1.2	42.5	[illegible]
Myanmar	13.85	2	0	45.35	4.74	53.04	2.2	-0.6	-1.2	34.4	[illegible]
Sudan	13.88	1	0	58.48	4.17	43.23	4.6	-1.4	-1.6	34.8	[illegible]
United Republic of Tanzania	13.97	2	0	49.31	2.66	59.87	4.9	-0.4	-0.8	39.5	[illegible]
Pakistan	13.97	2	0	67.35	4.70	223.29	3.6	-0.9	-0.7	29.8	[illegible]
Lao People's Democratic Republic	14.11	2	0	45.81	7.89	7.21	2.6	-1.1	-0.8	36.7	[illegible]
Timor-Leste	14.12	2	0	43.36	3.63	1.28	3.3			28.3	[illegible]
Turkmenistan	14.22	3	0	42.43	15.54	6.16	2.7	-1.4	-1.0		[illegible]
**Country**	**HINI**	**World Bank Income Classification**	**Modal AAD**	**U5MR**	**GDPpc**	**Population**	**Fertility Rate**	**Corruption**	**Gov Effectiveness**	**Gini Index**	[illegible]
China, Hong Kong SAR	3.33	4	90	2.02	59.59	7.51	1.1	1.7	1.7		[illegible]

© 2025 Bendavid E et al. *JAMA Health Forum*.
