# Supplementary material for: A Global Analysis of Within-Country Health Inequalities
Source: JAMA Health Forum. 2025 Oct 17;6(10):e253611. doi: 10.1001/jamahealthforum.2025.3611 (PMC12534847; doi:10.1001/jamahealthforum.2025.3611)
Supplement: Supplement 2. — Data Sharing Statement [file jamahealthforum-e253611-s002.pdf]

Data Sharing Statement

Bendavid. A Global Analysis of Within-Country Health Inequalities. *JAMA Health Forum*. Published October 17, 2025. doi:10.1001/jamahealthforum.2025.3611

Data
Data available: Yes
Data types: Data dictionary, Data (not involving human participants)
How to access data: We will create a public repository that will be available with publication
When available: With publication

Supporting Documents
Document types: Statistical/analytic code
How to access documents: We will create a public repository that will be available with publication
When available: With publication

Additional Information
Who can access the data: Public
Types of analyses: For any purpose
Mechanisms of data availability: Open
